# Ticket to Ride: I-deals as a Strategic HR Tool for an Employable Work Force

**DOI:** 10.3389/fpsyg.2021.769867

**Published:** 2021-11-22

**Authors:** Beatrice Van der Heijden, Aukje Nauta, Mel Fugate, Ans De Vos, Nikos Bozionelos

**Affiliations:** ^1^Institute for Management Research, Radboud University Nijmegen, Nijmegen, Netherlands; ^2^Faculty of Management, Open Universiteit Nederland, Heerlen, Netherlands; ^3^Department of Marketing, Innovation and Organisation, Ghent University, Ghent, Belgium; ^4^School of Business, Hubei University, Wuhan, China; ^5^Kingston Business School, Kingston University, London, United Kingdom; ^6^Department of Social, Economic and Organisational Psychology, Leiden University, Leiden, Netherlands; ^7^Department of Management and Information Systems, College of Business at Mississippi State University, Mississippi State, MS, United States; ^8^Next Generation Work expertise centre, Antwerp Management School, Antwerp, Belgium; ^9^Faculty of Business and Economics, University of Antwerp, Antwerp, Belgium; ^10^EMLyon Business School, Écully, France

**Keywords:** I-deals, employability, sustainable career perspective, integrative employment relationship, human resource management

## Abstract

We describe how idiosyncratic deals (I-deals), in this case I-deals focused on workers’ employability enhancement, can serve as a powerful strategic HR tool for simultaneously meeting both the strategic goals of employers and the career goals of employees. Building on a sustainable career perspective, I-deals are interpreted as highly valuable, as they can help individual employees to more easily adapt to the fast-changing environments that nowadays characterize society and the labor market. After theoretical outlines on the concepts of I-deals and employability, we argue that I-deals can form the basis for integrative employment relationships aimed at employability enhancement. This article concludes with concrete recommendations for practice, indicating that in order to enable the sound use of I-deals as a strategic HR tool, organizations should discuss I-deals and employability openly through constructive dialogue. Moreover, examples for achieving this through specific practices, such as working with employability coaches and world cafés on employability, are described.

## Introduction

*John worked in a call center of a public organization for several years and noticed that many callers asked the same questions over and over again. One day, John talked to his manager and said: “What if I were to make short movies of frequently-asked questions and post them on our website? After all, in my spare time I make training videos for our local soccer club and thus have the equipment and skills.” His manager, who happened to be a trusting leader said: “Go ahead.” Therefore, John did. Consequently, the number of phone calls, that both he and his colleagues, received decreased substantially, thereby significantly lowering the organization’s labor costs. Better still, John enjoyed his job much more now that he combined taking phone calls with making short video clips. After noticing the positive results, John’s manager offered to pay him to take a course in moviemaking, which John gladly accepted.*

This narrative illustrates how a tailored or special deal between a manager and an employee can produce positive outcomes for both. Rousseau (2001) referred to such arrangements as *idiosyncratic deals* or I-deals, which are mutually beneficial special arrangements negotiated between individual workers and their employers (Rousseau, 2005; Rousseau et al., 2006). I-deals are quite common and often made spontaneously without employees or managers being fully aware of the underlying constructive negotiation (i.e., mutually beneficial). Contributing to the value and appeal of I-deals is the fact they are often conceived of as classic win–win situations for the employee and employer. For instance, an employee might ask her manager if she can leave early on Thursdays to attend her evening MBA class. Both parties benefit from her increased knowledge and skills, and the employer facilitates this by allowing her to leave work early. As such, I-deal making is a pervasive phenomenon with a long but perhaps not well-documented history. Moreover, when done skillfully, I-deals for a specific employee need not produce detrimental consequences for team members. Triple-win situations may in fact result, wherein co-workers approve and also indirectly benefit from their colleagues’ I-deals (Lai et al., 2009; Bal and Rousseau, 2015). For example, the I-deal described at the start of this article led to a decrease in phone calls about the same questions, which made the work less routine, boring, and frustrating for both John and his peers.

That said, since scholars have turned their attention to I-deals and revealed their substantial value (Rousseau et al., 2006, 2016; Rosen et al., 2013), they have also found that surprisingly few employers and employees utilize them. Research in the Netherlands involving thousands of HR professionals shows that only one-third of Dutch employees actually make I-deals, and most of these work in small businesses (Van de Ven and Nauta, 2018). Similar results were found in a qualitative case study in the UK which showed that only 22% of employees in a public organization reported attempts to make an I-deal (Davis and Van der Heijden, 2018). This leads us and other scholars to strongly advocate a much broader and more strategic use by employees and employers across industries, both in small and large companies, as doing so can improve and help realize the full potential of employer–employee relationships.

Consistent with this position, and building on the sustainable career paradigm (De Vos et al., 2020; Van der Heijden et al., 2020), we are, to the best of our knowledge, the first scholars that approach the link between I-deals and employability, using this paradigm. In particular, we articulate how I-deals focused on enhancing the employability of an organization’s employees can serve as a practical strategic HR tool for meeting both the strategic goals of employers and the career goals of employees. As a strategic HR tool, employability-enhancing I-deals can include HR practices that help to align the objectives of the organization with the objectives of individual employees (*cf.* De Vos et al., 2015). In doing so, we take a multiple-stakeholder or systemic perspective (Colakoglu et al., 2006) and stress the need to adhere to the dual responsibility for protecting and further enhancing the worker’s career potential (i.e., their employability; Van der Heijde and Van der Heijden, 2006; Van der Heijden et al., 2018). To elaborate, as used here, we define employability as one’s ability to identify and realize career opportunities within and between organizations over time (Fugate et al., 2004, p. 23), and as “the ability to keep the job one has or to get the job one desires” (Rothwell and Arnold, 2007, p. 25). Van der Heijde and Van der Heijden (2006), in their competence-based approach to employability, stressed the added value of employability for objective and subjective career success (Gattiker and Larwood, 1986; De Vos et al., 2011) and defined the concept as “the continuous fulfilling, acquiring or creating of work through the optimal use of competences” (p. 453). As such, these competences are amenable to development and enhance job performance and employment opportunities. The premise is that I-deals that simultaneously meet employers’ strategic objectives and boost employees’ employability are mutually beneficial and worthy of consideration.

The main question motivating this article is: *How can employability-enhancing I-deals be used as a strategic HR tool?* We contend that this question is highly relevant for employers and employees alike given the intense competitive pressures in today’s global labor markets (Crook et al., 2011; Collet et al., 2015). Technological, political, and social factors act to accelerate changes in jobs, organizations, industries, and their associated labor market needs (Lawrence et al., 2015; Eichhorst et al., 2017). This, in turn, dramatically increases uncertainty and intensifies the need for employees and employers to adapt in order to survive and thrive. From the employees’ perspective, employability is now referred to by many as the contemporary form of job security (Fugate, 2006; Bernstrøm et al., 2019), as possessing and utilizing the competences outlined above are an individual’s means for obtaining, retaining, and developing employment opportunities within and between employers (e.g., Van der Heijde and Van der Heijden, 2006; Fugate and Kinicki, 2008). From the employers’ perspective, the tumultuous and ever-more competitive labor market necessitates alternative, innovative, and effective ways to attract, retain, and motivate talent (Trank et al., 2002). As such, employability can be seen as central to the employment exchange relationship (Fugate et al., 2021).

Moreover, people have to work longer, due to increasing life expectancies that have forced governments to increase retirement age (Vogel et al., 2017). Hence, as a result, people have to be life-long learners to keep up with constant changes even in their latest career stage (see for instance Pool et al., 2015; Oostrom et al., 2016; Le Blanc et al., 2017; De Lange et al., 2021). However, as people grow older, the way they learn inside and outside their job becomes more and more idiosyncratic, requiring unique and special agreements about learning opportunities (Froehlich et al., 2015). We start from a sustainable career perspective (De Vos et al., 2020; Van der Heijden et al., 2020) and argue that employability-enhancing I-deals can play an important role in protecting and further enhancing one’s health, happiness and productivity (being the core indicators of sustainable careers; *ibid.*; *cf.* Van der Heijden, 2005) throughout one’s career. A sustainable career perspective implies that careers are approached from the *individual perspective*, as the individual employee is perceived to be the central career actor. However, by addressing employability as a key element of the employment relationship, both individual and situational elements can be incorporated, thereby bringing in the needs and concerns of multiple stakeholders (*cf.* Colakoglu et al., 2006) in their *surrounding context*. In addition, the sustainability of one’s career comprises a *dynamic process,* as both employees and their context are prone to changes over time, thereby making it urgent to regularly investigate the suitability of a specific set of I-deals across different career stages (see also Demerouti et al., 2012).

In this contribution, we build on existing research and attempt to explicate the importance of employability-enhancing I-deals as practical, widely accessible and effective means for meeting the above-mentioned challenges (Oostrom et al., 2016; Davis and Van der Heijden, 2018; Rofcanin et al., 2018; Van Vianen et al., 2019). To this end, we first explain in detail what we currently know about I-deals and how they benefit employees, employers, and the relationship between the two.

## I-Deals and Associated Consequences

I-deals have been formally defined as “voluntary, personalized agreements of a nonstandard nature, negotiated between individual employees and their employers regarding terms that benefit each party” (Rousseau et al., 2006, p. 978). Negotiating special arrangements to meet an individual employee’s job and career needs is nothing new (e.g., Fried et al., 2007). For instance, the notion that job duties and acceptable work behavior are subject to negotiations between organizational members is a central assumption of organizational role theory, notably in Ilgen and Hollenbeck’s (1991) job role differentiation theory. Further, in previous research, the related construct of job change negotiation has already been examined as a proactive socialization tactic of organizational newcomers (Jones, 1986; Ashford and Black, 1996) and might therefore also be subject to I-deal negotiations.

However, and in contrast, what is new and increasingly valuable is making such negotiations more purposeful and strategic. This is where knowledge and application of idiosyncratic deals adds value. Unlike previous research and perspectives, the concept of I-deals is keenly focused on the personalizing of work, and as we advocate, on aligning employee and employer interests through individual negotiation and resource exchange, in order to simultaneously enhance employability and to meet organizational objectives. One reason for this is that the concept of I-deals is partly an outgrowth of research on psychological contracts—implicit agreements between employees and employers (Hornung and Rousseau, 2017)—which are often idiosyncratic employer–employee exchanges based on personalized terms and conditions (Schalk and Rousseau, 2001). I-deals are likely becoming even more prominent as a potential tool for personalizing the employment relationship in the post-pandemic era, where employers and employees are searching for ways to flexibly align their needs, as workplaces are opening again. Yet, employees may not want to return to the old collective agreements on working place and time, and the rich variety in individual needs might come to the forefront much more than before (Rudolph et al., 2021).

Another important feature of I-deals is their heterogeneous content (Rousseau et al., 2006), as they may refer to development opportunities (e.g., training and certification), tasks (e.g., assignments that fit individual skills or professional interests), flexible working hours (e.g., possibility of working from home), pay (e.g., individualized financial incentives) or specific solutions for personal problems (e.g., temporarily lowered work load to care for a sick child; see Hornung et al., 2010b; Liao et al., 2016, for more details). More specifically, I-deals are often distinguished in terms of content: development-related vs. flexibility-related. The former comprises learning and professional advancement and the latter comprises the distribution and scheduling of working hours (Liao et al., 2016; Hornung et al., 2018). However, some studies have included additional dimensions, such as reduced workload (Gascoigne and Kelliher, 2018) or customized job content (“task” I-deals— Hornung et al., 2010b). In a later study, Hornung et al. (2014) further differentiated within the category of development I-deals, resulting in a tripartite taxonomy of task, career, and flexibility I-deals. An alternative taxonomy of I-deals was proposed by Ng and Feldman (2015) who distinguished between six types of I-deals, specifically pay, advancement opportunities, training, career development, job security, and support with personal problems.

I-deals can also vary in scope, from a single idiosyncratic element in a standardized employment package to a completely idiosyncratic employment arrangement (Rousseau et al., 2006). Finally, I-deals differ in timing. Some are made *ex-ante* or before people begin a particular job (individualized terms in an employment agreement), but most are made *ex-post* or after working at a given employer for some period of time (Rousseau et al., 2016). Based on the assessment that they are generally more relevant, the majority of research has (explicitly or implicitly) focused on ex-post I-deals (*ibid.*). For instance, building on their review of the literature on valued resources that are commonly exchanged in employment relationships, Rosen et al. (2013) have developed a scale of ex-post I-deals that includes the four dimensions of schedule flexibility, location flexibility, task and work responsibilities, and financial incentives.

Notwithstanding their contribution to this scholarly domain, all of the above-mentioned scholars seem to agree about the fact that their respective taxonomies are not fully comprehensive, as the types of resources that can be subject to I-deals are abundant. This abundance was illustrated, for example, by the list of resources that Herriot et al. (1997) compiled in their study using a sample of 184 UK employees specifying what they expected from and were willing to provide to, their employers, as part of their psychological contract. Topics included training, fair HR procedures, consideration of family needs, consultation, discretion, humanity, recognition, a safe environment, fair rules, pay, benefits, job security, acceptable working hours, honesty, loyalty, treatment of organizational property, and flexibility. All these “resources” could possibly be the subject of an I-deal—as is shown in the overview of specific examples of I-deals (see Table 1)—because all of them could possibly be tailored to suit specific individual and organizational needs.

**Table 1:** Examples of I-deal types and their win-win outcomes.

I-deal example	Win for the organization	Win for the individual	Type of I-deal
An employee who experienced decreased job satisfaction got a project at another department. In this project, he could increase his knowledge and skills’ base. The growth in occupational expertise that she experienced enabled him to regain work energy.	Increased productivity	Increased job satisfaction	Challenge
An employee who is a fervent surfer is enabled to take a day off whenever it blows really hard, and to work really hard and during a long day in the weekend (no interruptions and no commuting time) to pay it back, when timing of the deadlines allow him doing so.	Increased commitment	Increased job satisfaction	Comfort
A nurse, who makes work schedules for the whole department, has agreed with her manager to perform this task from home. In doing so, she saves 1.5h commuting time each day and can also provide care for her ill mother who lives with her, at the start of the day.	A self-managing team with regard to work schedules	Being able to concentrate on a task that needs focus and to combine this with family care	Comfort
An employee, who wants to become project manager, was given the opportunity by his manager to lead a large project, and herewith to increase the amount of responsibilities in his job.	Opportunity to assess somebody’s growth competencies on-the-job	Career advancement	Challenge

Although the specific subjects covered by I-deals can vary widely, a main distinction between different types of I-deals might be the degree to which they either *comfort* people—i.e., making work and working conditions somewhat easier—or *challenge* them—i.e., making work and working conditions somewhat more demanding (Nauta and van de Ven, 2015). Indeed, all of the I-deal distinctions listed in Table 1 fall into one of these two categories. For example, an I-deal on development challenges people, whereas an I-deal on working from home comforts people in combining their work and private life better. I-deals may be a logical answer for organizations to the fast-changing environments that characterize todays’ society and labor market (Jagannathan et al., 2019), as they can enable individuals to adapt to a new situation more easily than under large-scale standardized policies and procedures. I-deals have been shown to relate to individual employee commitment (Lemmon et al., 2016), productivity (Vidyarthi et al., 2016) and, especially as part of an integrated HR system, to organizational-level performance (De Vos and Cambré, 2017). However, beyond commitment, productivity, and performance benefits, which are mostly to the advantage of employers, I-deals also offer benefits for the individual employees who enter such deals. For example, research by Hornung et al. (2018) showed that I-deals about flexible working hours—which fall into the category of comfort I-deals—are related to lower work–family conflict. This kind of comfort I-deals can also increase the motivation of older workers to keep on working after retirement (Bal et al., 2012).

Furthermore, I-deals have been shown to relate to employees’ self-enhancement (Liu et al., 2013). As such, they represent an opportunity for individuals to develop their unique talents and motivations, in such a way that they can reach their fullest potential and deliver maximal value to personal, organizational and societal goals. However, research on I-deals is still relatively nascent, in particular with regard to longer-term consequences, such as workers’ employability, a key feature in contemporary employment relationships (Hillage and Pollard, 1998; Forrier and Sels, 2003; Fugate et al., 2004; Van der Heijde and Van der Heijden, 2006; Rothwell and Arnold, 2007; Fugate et al., 2021). Therefore, after an outline on the notion of employability as a strategic issue for todays’ companies, we will look into the added value of I-deals as the basis for integrative employment relationships, which are those that explicitly account for the needs and interests of both the employer and the employee.

## Employability and Strategic HRM

Employability or career potential (Van der Heijde and Van der Heijden, 2006; Van der Heijden et al., 2018) refers to the ability of workers to perform their current job and to acquire or create a new job by making optimal use of their personal competences (Van der Heijde and Van der Heijden, 2006, p. 453). Defined as mutually beneficial, I-deals are assumed to generally exert a positive influence on workers’ employability, although this does not rule out differential effects of specific types of I-deals, as well as potentially undesired side effects. For instance, having a unique deal with an organization might bind a certain employee to his or her employer, thereby decreasing their external employability (Kroon et al., 2018). Therefore, we posit that the relationship between I-deals and employability warrants a theoretical analysis to clarify several conceptual distinctions and boundary conditions. As already described above, until the 1990s, most work could be done in largely standardized jobs. However, the rapid pace of technological, economic and social developments in the contemporary labor market implies that organizations can no longer rely on people doing the same jobs for years (Fugate et al., 2021). At the same time, the professional skills, innovativeness and commitment of their workforce, as well as their capacity to adapt to ever-changing circumstances, are becoming more and more critical for organizations to perform and stay competitive (Kostopoulos et al., 2015; Apenko, 2017). The qualities of today’s workforce that are needed to enable the organization to adapt and survive in today’s environment underlie current employability requirements (Van der Heijde and Van der Heijden, 2006; Thijssen et al., 2008; Fugate et al., 2021). Building on Fugate et al. (2021, p. 291), we argue that, in order to prevent the risk of intensifying the growing skills gap and Matthew effect (rich get richer and poor poorer), a multiple-stakeholder approach is crucial. In applying this, the needs of employees and employers should be aligned, while also integrating these with formal and informal training, vocational, and governmental policies and practices.

A case in point is the HR vision of Unilever, a multinational company that recently had the following posted on its website: “We believe our employees are not defined by their job titles, but by the positive impact they make through the work they do.” And: “Be yourself. At Unilever, we do not just say it, we celebrate it! Unilever employees are not just comfortable being themselves at work, they are encouraged to do it. Here, you do not need to change to be successful. Our leadership plays a vital role in ensuring that everyone is valued, included, and brings his or her personal passion into the workplace. We work hard to build a strong sense of community by tapping into the thoughts and experiences of every Unilever employee. Through this, we foster an inclusive culture and provide leadership, professional development, and networking opportunities.^1^”

Of course, these are marketing messages to attract talented employees, but at least the HR message of Unilever, and many other companies today, is in line with the idiosyncratic approach proposed by Van der Heijden (2005; see also Van der Heijden and De Vos, 2015). Specifically, Unilever respects their employees’ personal preferences, needs, capabilities, and individual aspirations along the life span. In other words, they want to adapt to individual talents and needs and support bottom-up approaches of HR practices and individual arrangements, thus utilizing I-deals that build on an integrative employer–employee relationship. Such I-deals are intended to foster and enhance employability and complement the traditional top-down implementation of HRM.

## I-Deals, Employability-Enhancing I-Deals, and Integrative Employment Relationships: Theoretical Considerations

Individual arrangements may be the key to an employable workforce, especially when employees and their managers make I-deals about learning, development and innovation. In these kinds of challenge I-deals, both the employer and the employee thrive. The employer, because they can use talents and competencies of the employee which would not have been apparent if the employee only performed a standardized job. In addition, the employee, because he or she can develop talents and competences in directions that meet their needs and desires. Hence, we conceptualize I-deals as the smallest building block for *integrative employment relationships*, i.e., employment relationships in which the needs and interests of both the employer and the employee are met. In other words, we argue that I-deals are by definition win–win. However, at the same time, we posit that beneficial effects of I-deals cannot be taken for granted. They are tied to certain preconditions (Rousseau, 2001; Rousseau et al., 2006). For instance, Lai et al. (2009) showed that co-worker acceptance of the I-deals of their peers is influenced by the quality of the employment relationship, by the interpersonal relationship with the I-dealer, as well as by the confidence of the person to be able to obtain a similar special arrangement if needed. Likewise, a more recent pilot study on employee attitudes, toward the legitimacy of I-deals as a management practice, found that such personalized arrangements were generally judged to be fairer when they were widely available, and when the organization adhered to principles of procedural justice (Hornung et al., 2016).

Moreover, from earlier empirical work it is known that not all types of I-deals have the same outcomes. In order to better understand the possible impact of I-deals, we posit that social exchange theory (Blau, 1964; Cropanzano and Mitchell, 2005) provides a valuable lens and may serve as an underlying theoretical mechanism to understand the linkage between I-deals and employability (*cf.* Fugate et al., 2021). As the notions of interdependence and reciprocity lie at the heart of social exchange theory (Cropanzano and Mitchell, 2005), Fugate and associates (2021) urge scholars in this domain to adopt an approach of concurrent consideration of perspectives to gain more insight into how both parties (employer and employee) bring their own interests to the employment relationship, and how these interests can be aligned (p. 282). The Strategic Employability Architecture (SEA) framework, that they have created, is meant to guide scholars on how to approach the interdependent employer–employee relationship. Their SEA framework is an adaptation of the human resource architecture developed by Lepak and Snell (1999, 2002), and may help us to further our understanding of which I-deals are particularly relevant to increase a worker’s employability, herewith taking into account the specific nature of the employee–employer relationship (see Fugate et al., 2021 for more details) and therefore differentiating according to the needs of specific categories of workers too.

More specifically, some I-deals are more likely than others to increase employability. Certain challenge I-deals, for instance, may provide an employee exposure and opportunities to work in an area of higher strategic importance to their employer than her/his typical position. As an example, many organizations around the globe are implementing artificial intelligence in various functional areas, such as operations and human resources. If an employee seeks (proactive) or accepts (reactive) an opportunity to build their knowledge, skills, and abilities, then they will presumably enhance their employability both inside and outside their employer (Fugate et al., 2021). Such opportunities may also increase an employee’s sense of value and satisfaction (psychological benefits), as well as additional and perhaps more rewarding relationships (social benefits). More generally, challenge I-deals, like the one described, provide the possibility of expanding an individual’s competencies, which in turn is likely to increase their perceived value in the “eyes of the employer” and enhance their employability. These assertions are supported by research that shows that employability enhancement is associated with promotability by supervisors and perceptions of greater long-term opportunities by employees (Oostrom et al., 2016).

Challenge I-deals that are employability-enhancing are also supported by research related to continuous learning and employability (*cf.* the impact of the learning value of the job; Van der Heijden and Bakker, 2011). Challenging assignments not only require an employee to expand the breadth and complexity of their competencies, but they also often include greater responsibilities (Srikanth et al., 2020) and enhanced opportunities for learning through experience (Dragoni et al., 2009; Seibert et al., 2017). Research related to self-enhancement theory and I-deals (Korman, 2001) suggests that such experiences can enrich one’s self-evaluations, which in turn may boost one’s motivation for additional growth opportunities and the approval of others (Korman, 2001). Although not tested, this research and reasoning suggests the potential for a positive psychosocial employability-enhancing feedback loop or spiral (*cf.* Fugate et al., 2021).

On the contrary, comfort I-deals might *decrease* an individual’s employability. For example, if a worker gets the opportunity to work fewer hours due to issues at home, this may imply a short-term solution that is highly comfortable for him or her, and that also fits the organization. Yet, in the end, the worker’s employability might be impaired due to the fact that he or she is less visible (see for instance Lawrence and Corwin, 2003; Rousseau et al., 2016; Ng, 2017). For yet another employee, who is facing the risk of burnout due to more severe family circumstances, the exact same type of I-deal might rather protect his or her work-life balance, and through this, his or her employability in the long run (see also Las Heras et al., 2017). However, other I-deals might have no relationship with employability, for example the I-deal of bringing a dog to the office, although, in this particular case, we might even hypothesize on the protective effect, in terms of health and psychological well-being of having a pet (see for instance Herzog, 2011). As noted above, a particular I-deal may have different effects (comfort and/or challenge), depending on an individual’s specific circumstances (Nagy et al., 2018; De Vos et al., 2020). We argue that over time both types of I-deals need to be considered.

In addition, we suggest that the impact of I-deals on employability partly depends on the extent to which the underlying negotiation process was based on a distributive or integrative dialogue. A sound negotiation process implies that I-deals are based on a mutual agreement between employer and employee. In this negotiation process, individual employees commonly bargain for themselves, while the employer is represented by an agent in the organization who possesses legitimate authority (such as higher-level managers or HR representatives) to grant I-deals (Hornung et al., 2010a). The direct supervisor of the employee, in particular, appears to play an important role as negotiation partner for I-deals (e.g., Hornung et al., 2008). In distributive negotiations, desired outcomes are more or less equally allocated to employee and employers, whereas in integrative negotiations, the interests of both parties are addressed concurrently, often in creative and novel ways (see for instance, Janssen et al., 1999; Simosi et al., 2021).

Overall, I-deals are the result of bilateral interpersonal negotiation processes between individual employees and their employer, where the latter is typically represented by organizational agents with the authority to legitimize such special arrangements, which are not part of a standardized policy, collective agreement or HR package. Although direct supervisors most often fulfil the role of bargaining partners for employees, I-deals can also involve negotiations with HR representatives or higher-level management. Rousseau (2005) provides examples of such complex constellations, such as an engineer desiring re-assignment to London from Chicago, for which she had to negotiate, not only with her direct manager but also with her manager’s boss and the HR manager responsible for handling expatriate assignments (Rousseau, 2005, p. 37–38).

Although early conceptual work on I-deals provides examples and anecdotal evidence, research on the negotiation processes through which these personalized arrangements are created has been limited to interpersonal factors that may (or may not) lead to I-deals. An exception is the scholarly work by Ho and Tekleab (2016) that aimed to disentangle the relationship between the request of I-deals and the receipt of these I-deals. They found that the positive relationship between requesting and receiving I-deals was stronger where there were higher levels of Leader–Member eXchange (LMX). In addition, they found that receiving I-deals was related positively to employee’s job satisfaction and affective commitment. Conversely, the relationship between receiving I-deals and turnover intention appeared to be negative. In a similar vein, Kong et al. (2020), in their study using 131 co-worker dyads, demonstrated the interpersonal implications of task I-deals for emotional exhaustion and subsequent deviant behaviors.

We argue that the process of negotiation needs serious attention in order to enhance our understanding of I-deal making, as not only the outcome of the process but also the process in itself is likely to have an impact on subsequent employee outcomes. For instance, when the negotiation process leading to an I-deal has taken place in a negative climate, even though in the end the I-deal is obtained, the negative experience with this process might reduce the potential beneficial effects of the I-deal on the worker’s commitment or performance. Corroborating this view, the negotiation literature already reported that there is no one way of negotiation; instead, there are at least five styles that negotiation parties can use, running from forcing, giving in, avoiding, compromising, to problem solving (De Dreu, 2010). These styles might have a differential impact upon the attitudes and behaviors of the negotiation parties involved, following the negotiated agreement (Pruitt and Carnevale, 1993; De Dreu and Weingart, 2003).

## I-Deals, Employability Enhancing I-Deals, and Integrative Employment Relationships: Practical Considerations

In practice, whether or not an employee and his or her manager is able to close an I-deal depends upon the negotiation process that follows the proposition of an I-deal by either the employee or the agent. Given that, by definition, I-deals are meant to serve the interests of both parties (Rousseau, 2005), a first precondition is that the negotiations that characterize these should be of an integrative nature (see Table 2). Integrative negotiations are common when there is already some kind of relationship between the parties involved, and that the parties would like to preserve.

**Table 2:** Preconditions for I-deals.

Preconditions for I-deals
The I-deal negotiations should be of an integrative nature
It is crucial that these parties acknowledge that their I-deal negotiations produce greater benefits for each
I-deals need to be accessible for all, and not only for the ‘happy few’
I-deal making needs a climate of high trust and high commitment

Second, it is crucial that these parties acknowledge that their negotiations produce greater benefits for each, rather than each considering only their own interests (McKersie et al., 1965). On the other hand, distributive negotiations occur when parties perceive a zero-sum game, where each can benefit only if they seize something from the other party. This type of negotiations is common in situations wherein there is no prior relationship between the parties involved nor a belief by any of the parties that it is important to cultivate some kind of relationship for the future.

A third precondition under which I-deals may serve the employability of all kinds of workers is that they need to be accessible for all, and not only for the “happy few” (*cf.* Fugate et al., 2021). Unfortunately, this precondition is often not met. In particular, it has been shown that some employees are more likely than others to manage to close an I-deal. For example (Van de Ven et al., 2018) reported that those employees who have already obtained good assessments by their managers are more likely to make an I-deal in comparison with their colleagues who obtained poorer assessments (*cf.* the notion of resource gain spirals in Conservation of Resources theory; Hobfoll et al., 2018 and *cf.* Figure 2, Page 286 from Fugate et al., 2021).

A fourth and final precondition is that I-deal making needs a climate of high trust and high commitment, as in high commitment or high-performance systems (HPWSs; Pfeffer and Veiga, 1999; Appelbaum et al., 2000). A HPWS is a system that reflects HRM that is focused on investments in people (Jewell et al., 2020). HPWSs emphasize, among others, systematic training and development of employees, job variety and skill flexibility, opportunities for internal mobility and promotions, employee autonomy and involvement in influencing work processes, and job security (Subramony, 2009; Posthuma et al., 2013). Employers who make use of HPWSs tend to have a long-term interest in their workforce and *vice versa* (Pfeffer and Veiga, 1999; Karadas and Karatepe, 2019).

Employers with a strong HPWS orientation can be viewed as having a strong interest in the long-term development and retainment of their staff members (Pfeffer and Veiga, 1999). Within organizations that emphasize the HPWS approach, employees will feel more secure and motivated to initiate discussions about I-deals as, under such circumstances, organizational agents will be more open and willing to engage creatively in such discussions. A crucial requirement for creative dialogues is a focus on topics that are relevant and meaningful for both the employee and the organizational agent (Gratton and Ghoshal, 2002), because both negotiating parties need to feel personal engagement with the specific topic under discussion, in order to be willing to look for integrative solutions. The HPWS approach is closely related to employability; the more extensive the utilization of HPWSs, the more likely that the long-term employability of the workforce is enhanced. From an employability point of view, an employee needs an employer who appreciates and invests in the employability of its workforce, while the employer needs employable people in order to achieve constantly changing organizational goals in this dynamic, global world wherein career sustainability is key (De Vos et al., 2020; Van der Heijden et al., 2020; Fugate et al., 2021). In the next section, we will look into the use of I-deals as strategic HR tools that are intended to protect and further enhance workers’ employability, and organizations’ competitive advantage.

## How to Use I-Deals as a Strategic HR Tool

How can I-deals be used as a strategic tool to achieve the common interests of employers and employees, including fostering the career sustainability of employees? On the one hand, one could argue that I-deals “just happen” in trustful working relationships between employees and managers who want to make the most out of their collaboration. Indeed, I-deals often arise spontaneously, during conversations in which employees and managers search for solutions for everyday challenges at work. On the other hand, when employees and managers are fully aware of the potential benefits of I-deal making, they will know how to use I-deals strategically, for example during formal yearly conversations in which not only individual performance is assessed, but also future plans are made. The question is then: What policies and practices can an organization implement, in order to increase the likelihood that employees and managers will deliberately engage in I-deal making? Based on what we have experienced during consultancy projects in different organizations, we have three concrete recommendations for practice.

First, organizations may want to write in their Collective Labor Agreement (CLA) or personnel handbook that they openly encourage, and give all employees and managers the opportunity to negotiate I-deals. ING Bank in the Netherlands does so openly in their “CLA 2019-2020”, by writing: “In the new CLA entitled ‘Our happiness at work’ the parties to the CLA want to contribute to your professional happiness. That is why we are providing employment conditions with fewer rules and more principles, meaning frameworks and agreements that are aligned to your wishes, that recognize your talent and that are a good fit within the Accelerated Think Forward strategy”.^2^ Furthermore, in their CLA, ING introduced and described a so-called Unlimited leave pilot, in which they leave it up to the individual employees and their managers to decide when, and for how long, an employee wishes to take leave, as long as they fulfill their tasks satisfactorily within the agreed upon time frames. If the pilot is successful, it will be implemented as a policy for all employees in the next CLA. Finally, the CLA writes: “The automatic coupling of performance management and reward will be discontinued. This will enable us to create room to engage in open dialogue about your development without this having any implications on your salary.” In sum, ING encourages their employees and managers to arrange their own idiosyncratic working relationships, as a strategy to stimulate happiness at work together with craftsmanship, goals that satisfy the needs and interests of employees and the company alike.

Second, an organization can work with so-called employability coaches: professionals to whom employees can turn for help with specific work and career challenges. This is what Rijkswaterstaat does, a Dutch government organization responsible for the design, construction, and maintenance of the main infrastructure facilities in the Netherlands (Van de Ven and Olivier, 2019). The employability coaches have consultation hours where employees can easily walk in to talk about work, vitality and career problems. These conversations help employees to gain new ideas for boosting their employability. For example, one employee thought of moving to another department, although she was still satisfied with her current job. During the conversation with the employability coach, she figured out that she might already start working in that other department for one day a week. Next, she talked things through with her manager and with the manager of the other department. They were both enthusiastic about her idea. This I-deal was soon drawn up. This example shows that employability coaches can help employees to prepare for a dialogue with their manager in which they can achieve an I-deal.

Finally, companies can organize so-called world cafés for their employees: a structured conversational process in which several groups of four to eight employees talk about their employability issues at small tables like those in a café. Questions are pre-defined—sometimes even printed out on a tablecloth—in order to guide and structure the conversation. During the conversation, employees give and receive help from each other on how to gain ideas for i-deals that might increase their employability. Philips, a multinational electronics and health technology company, organized several of these world cafés and succeeded in making their employees enthusiastic about i-deals and employability.^3^ In sum, in order to enable the sound use of i-deals as a strategic hr tool, organizations should address the importance of dialogue, i-deals and employability openly in their collective labor agreement, personnel handbook or other strategic hr documents and implement specific practices, such as employability coaches and world cafés on employability, and through this help their employees to prepare for i-deal making with their manager. Preferably, they do so by departing from a sustainable career perspective (De Vos et al., 2020; Van der Heijden et al., 2020), hereby aligned employability-enhancing i-deals to the individual employee’s needs across different career stages (Demerouti et al., 2012) in order to do justice to the highly idiosyncratic nature of careers and their constantly changing dynamics. We advocate considering both comfort and challenge i-deals, given the large variety in inter- and intra-individual employability changes over time, resulting into a pluriformity In employees’ needs, that are related to their organizational, functional, psychological or life-span development age, over and above their chronological age (Sterns and Doverspike, 1989; see Also Le Blanc et al., 2017).

## Next Steps for Researchers

Our overarching goal was to inspire scholars in this field and foster new research on the link between I-deals and employability. We call for more research wherein the three dimensions of sustainable careers—person, context, and time (De Vos et al., 2020; Van der Heijden et al., 2020) —are studied in tandem. This will shed more light on the added value of employability-enhancing I-deals in the light of employees’ health, happiness, and productivity. Building on the notion that I-deals form the basis of integrative employment relationships, we recommend examining both employee and employer outcomes simultaneously. In a similar vein, we advise future researchers to operationalize the indicators of sustainable careers by means of a broader set of variables (for instance both physical and mental health, work engagement, and performance, to mention just a few), and by using multiple measurement waves, hereby allowing a better understanding of possible interactions between these variables, over time. To achieve this, it will be necessary to examine relevant factors over both short and long time intervals, as employability and career development include both macro- and micro-changes in predictors over time (Van der Heijden et al., 2020). We therefore recommend adopting both variable-centered and person-centered approaches (Laursen and Hoff, 2006; Morin et al., 2018), to identify different trajectories of (un)sustainable careers and the possible impact of I-deals in this regard. It would also by highly beneficial to examine intra-individual changes in perceived employee–employer relationships (e.g., psychological contracts) over time in relation to the prevalence of employability-enhancing I-deals and their impact on the employee’s career sustainability.

More research is also needed to investigate how employability-enhancing I-deals may protect career sustainability when the individual employee experiences a career shock. A career shock is “a disruptive and extraordinary event that is, at least to some degree, caused by factors outside the focal individual’s control and that triggers a deliberate thought process concerning one’s career” (Akkermans et al., 2018, p. 4). The pandemic caused by the COVID-19 virus can be conceived as a particular career shock in this regard (Akkermans et al., 2020), which has spurred unforeseen changes in ways of working, especially when it comes to teleworking. It would be interesting to investigate *via* longitudinal research how employers and employees are re-defining the employment relationship and whether the massive switch to teleworking will give rise to increased opportunities for negotiating I-deals on worktime and place as workplaces open up again.

In addition, we call for more research on the link between I-deals and employability herein differentiating according to employee’s age, gender, and working time (part-time vs. full-time) and according to organizational size, as previous research has indicated that these factors can relate to an employee’s actions to negotiate for I-deals (Ragins et al., 2000; Hornung et al., 2008; Bal et al., 2012; Rosen et al., 2013; Lee et al., 2015; Liao et al., 2016; Tuan, 2016; Bal and Boehm, 2019). Last but not least, given the demise of the public sector’s notion of a “job for life,” special scholarly attention is also needed for public organizations, wherein one investigates how I-deals may help to accept the changing psychological contract (Hiltrop, 1995), and through this, to better understand its link with employability (*cf.* Davis and Van der Heijden, 2018).

We hope that approaching I-deals as the building blocks of integrative employment relationships will guide scholars in the field to advance research on the linkage between I-deals and employability and will hereby add to the career sustainability of individual workers as well as to the competitive advantage of organizations.

## Author Contributions

BH and AN: conceptualization, theorizing, writing original draft, and review and editing. MF, AV, and NB: theorizing, writing original draft, and review and editing. All authors contributed to the article and approved the submitted version.

## Acknowledgments

We would like to acknowledge Severin Hornung and thank him for his critical reflections on an earlier version of the paper.

## Conflict of Interest

The authors declare that the research was conducted in the absence of any commercial or financial relationships that could be construed as a potential conflict of interest.

## Publisher’s Note

All claims expressed in this article are solely those of the authors and do not necessarily represent those of their affiliated organizations, or those of the publisher, the editors and the reviewers. Any product that may be evaluated in this article, or claim that may be made by its manufacturer, is not guaranteed or endorsed by the publisher.

## Footnotes

^1^ https://careers.unilever.com/nl/en/people-with-purpose-thrive

^2^ https://www.fnv.nl/getmedia/7128b5ef-1860-40ce-a285-c8aa347e4fd2/1036-ING-CLA-2019-2020-v-011119.pdf

^3^ https://www.dekoers.nl/cases/philips-werk-en-toekomst/
